# Identification of copper metabolism and cuproptosis-related subtypes for predicting prognosis tumor microenvironment and drug candidates in hepatocellular carcinoma

**DOI:** 10.3389/fimmu.2022.996308

**Published:** 2022-10-06

**Authors:** Xianglong Liu, Bo Sun, Yiyang Yao, Linying Lai, Xueyuan Wang, Jie Xiong, Xiaoan Zhang, Jie Jiang

**Affiliations:** ^1^ Department of Radiology, Zhengzhou Central Hospital Affiliated to Zhengzhou University, Zhengzhou, China; ^2^ Department of Gastroenterology and Hepatology, Institute of Digestive Disease, Tongji Hospital, Tongji University School of Medicine, Shanghai, China; ^3^ Department of Gastroenterology, Qidong People’s Hospital, Nantong, China; ^4^ Department of Radiology, The Third Affiliated Hospital of Zhengzhou University, Zhengzhou, China; ^5^ School of Clinical Medicine, Henan University of Science and Technology, Luoyang, China; ^6^ National Center for Liver Cancer, Second Military Medical University, Shanghai, China

**Keywords:** hepatic carcinoma, cuproptosis, copper metabolism, tumor immune microenvironment, prognosis, therapy

## Abstract

Copper (Cu) is an essential element of organisms, which can affect the survival of cells. However, the role of copper metabolism and cuproptosis on hepatic carcinoma is still unclear. In this study, the TCGA database was used as the test set, and the ICGC database and self-built database were used as the validation set. We screened out a class of copper metabolism and cuproptosis-related genes (CMCRGs) that could influence hepatic carcinoma prognosis by survival analysis and differential comparison. Based on CMCRGs, patients were divided into two subtypes by cluster analysis. The C2 subtype was defined as the high copper related subtype, while the C1 subtype was defied as the low copper related subtype. At the clinical level, compared with the C1 subtype, the C2 subtype had higher grade pathological features, risk scores, and worse survival. In addition, the immune response and metabolic status also differed between C1 and C2. Specifically, C2 subtype had a higher proportion of immune cell composition and highly expressed immune checkpoint genes. C2 subtype had a higher TIDE score with a higher proportion of tumor immune dysfunction and exclusion. At the molecular level, the C2 subtype had a higher frequency of driver gene mutations (TP53 and OBSCN). Mechanistically, the single nucleotide polymorphisms of C2 subtype had a very strong transcriptional strand bias for C>A mutations. Copy number variations in the C2 subtype were characterized by LOXL3 CNV gain, which also showed high association with PDCD1/CTLA4. Finally, drug sensitivity responsiveness was assessed in both subtypes. C2 subtype had lower IC50 values for targeted and chemotherapeutic agents (sorafenib, imatinib and methotrexate, etc.). Thus, CMCRGs related subtypes showed poor response to immunotherapy and better responsiveness to targeted agents, and the results might provide a reference for precision treatment of hepatic carcinoma.

## Introduction

Liver cancer is one of the most common malignant tumors and the fourth leading cause of cancer-related deaths worldwide, threatening human health and life seriously (1, 2). Hepatocellular carcinoma (HCC) is a common type of liver cancer, accounting for almost 90% of primary liver cancers (3, 4). Some risk factors have been reported to be involved in HCC development, thereinto, excessive alcohol intake and virus infection, including hepatitis B or C, and Nonalcoholic fatty liver disease (NAFLD) are the leading risk factors (5–8). The new therapeutic tools and strategies for HCC, such as surgical treatment, radiotherapy, transplantation, and chemotherapy, have been developed in recent decades (9). However, multiple factors, such as tumor heterogeneity, high recurrence rate and metastasis, contribute to the low survival rate of HCC (10). Therefore, it is necessary to investigate in detail at the molecular level to improve the treatment efficacy for HCC.

Copper is an essential nutrient element for life involved in various biological processes, including cell proliferation, growth and metabolism. Cells regulate copper homeostasis through absorption, transport, uptake and storage to achieve an active copper balance. Alterations of copper levels may also lead to cytotoxicity and affect the development and progression of cancer (11, 12). Previous studies presented that the copper content in cancer patients is higher than that in healthy counterparts (13–15). Interstingly, latest study showed copper overload also cause a new form of cell death termed cuproptosis, which is different from apoptosis, pyroptosis, necroptosis and ferroptosis. Cuproptosis happens on that copper directly binds to lipoylated proteins in the tricarboxylic acid (TCA) cycle, which causes the acuteproteotoxic stress, the dysfunction of mitochodrial metabolism and, ultimately, cell death (11, 16). Thus, The role of copper metabolism and cuproptosis in tumorigenesis and progression deserves further attention.

It is commonly accepted that tumor microenvironment (TME) acts as a critical role in tumor development and progression (17). TME contains various cell types, such as immune cells, fibroblasts, endothelial cells, and a variety of soluble substances, including inflammatory factors, cytokines, chemokines (18). Then, TME affects the malignant cells through soluble substances and extracellular matrix elements, which promote tumor progression and predict prognosis. Emerging evidences presented that overload of copper, the intermediates of copper metabolism and the production of cuproptosis led to immune dysfunction through reactive oxygen species (ROS). For example, ROS could contribute to the damage-associated molecular patterns (DAMP) release, which significantly regulates immune response (19, 20). However, the relationship between copper metabolism and cuproptosis-related genes (CMCRGs) and prognosis of HCC patients is not clear. Thus, the molecular characteristics of CMCRGs may provide important insights to understand the characteristics of TME and the underlying mechanism of HCC, then predict the immunotheray and prognosis.

In this study, we analyzed expression of CMCRGs in HCC from TCGA, and then univariate Cox was used to screen out 49 CMCRGs, which have significant effect on the survial of HCC patients. Furthermore, we established molecular subtype of HCC based on these genes related to copper metabolism and cuproptosis, and verified the stability of the subtype using self-constructed database and International Cancer Genome Consortium (ICGC) database. Next, we analyze the TME, somatic mutation landscape, tumor mutation burden (TMB), immune checkpoint genes (ICGs), and treatment sensitivity between two different subtypes. In summary, we demonstrated the molecular subtype of HCC based on CMCRGs, which predicts prognosis for HCC patients, and provides new insights for treatment.

## Materials and methods

### Data collection and processing

**Figure 1:**
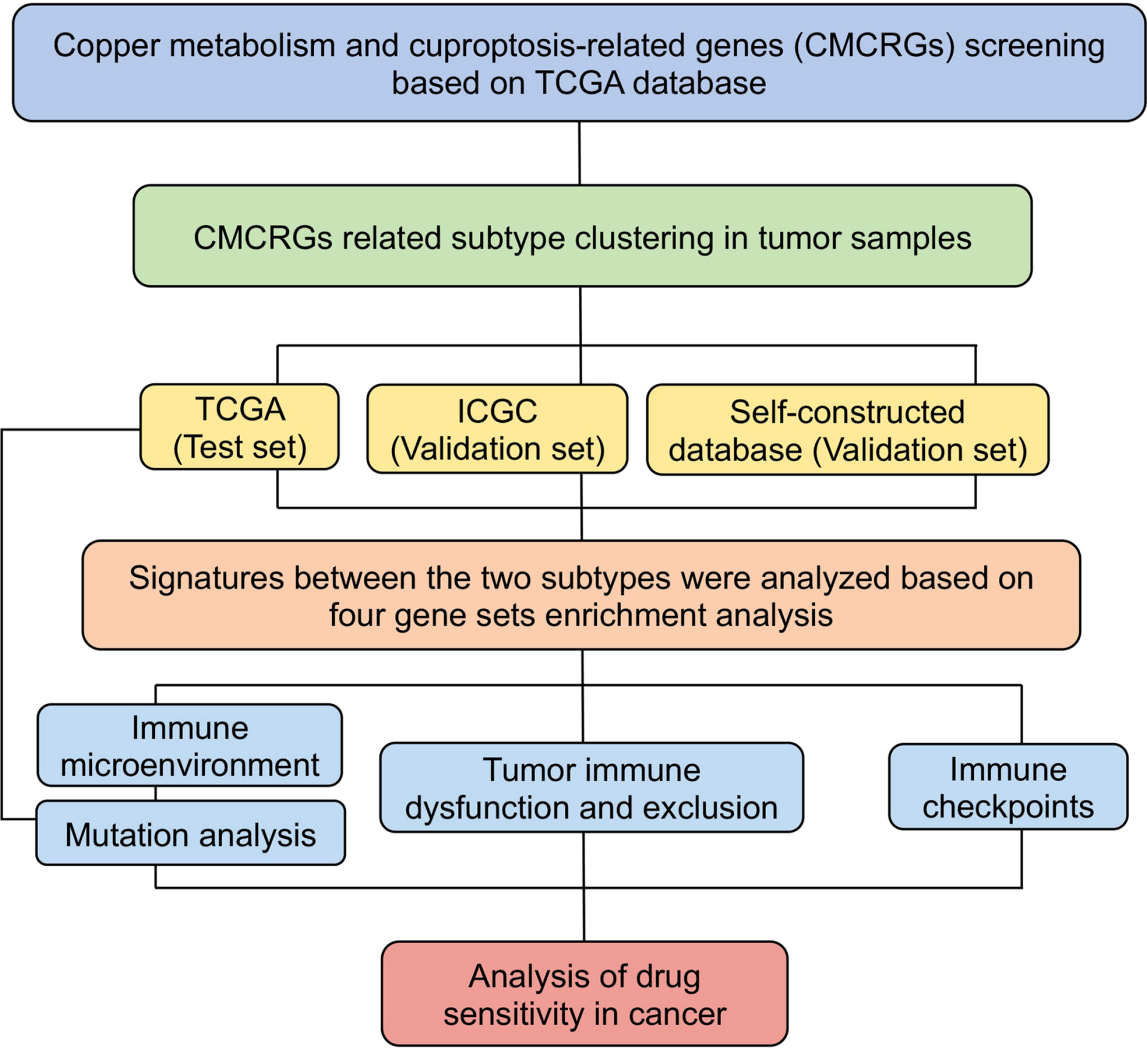
Flow chart of this study.

The process used for analysis is summarized in the flow chart (**Figure 1**). The transcriptional profiles, clinical follow-up information (including age, sex, TNM stage, tumor grade, survival time, and survival status), single nucleotide variation (SNV) and copy number variation (CNV) data of HCC patients were downloaded from the liver hepatocellular carcinoma (LIHC) cohort in The Cancer Genome Atlas (TCGA) database (https://portal.gdc.cancer.gov). The data from self-constructed database and International Cancer Genome Consortium (ICGC) database (https://dcc.icgc.org/) were used as validation sets (**Table S1**). The self-constructed database was 57 pairs of cancer and adjacent tissues diagnosed as hepatocellular carcinoma by biopsy were sequenced for gene expression profile by Illumina platform. We finally acquired a total of 50 normal and 374 HCC samples from the above publicly available open data sources. A total of 137 CMCRGs were searched from Molecular Signature Database v7.5.1 (MSigDB, https://www.gsea-msigdb.org/gsea/msigdb/) and previous literatures, which are summarized (**Table S2**).

### Differentially expressed CMCRGs

To identify the CMCRGs involved in the progression of HCC, we performed differentially expression analysis comparing the 50 normal samples and 374 tumor samples in TCGA-LIHC cohort. Differentially expressed genes (DEGs) were screened out with the threshed set to the |log2 fold change (FC)| > 0 and *P* value < 0.05 using the R package “edgR”. To further explore the biological function of these significant DEGs, GO and KEGG enrichment analyses were conducted using the “clusterprofiler” package in R.

### Identification of overall survival associated CMCRGs

To understand the potential prognostic significance of CMCRGs in HCC, we investigated the overall survival (OS) associated CMCRGs using univariate Cox proportional hazard regression analysis in TCGA-LIHC (n = 370). *P* values < 0.05 were considered statistically significant, and all *P* values correspond to two-sided significance tests.

### Consensus clustering analysis of CMCRGs

Consensus clustering analysis is a robust unsupervised classification technique achieved through multiple resampling and clustering, which was performed to classify HCC patients into distinct molecular subtypes according to CMCRGs expression using the ‘ConsensusClusterPlus’ package. K-means algorithm and cumulative distribution function (CDF) curve were used to determine the best number of subtypes, and 50 iterations with maxK=9 were carried out for stable subtypes.

### Relationship between molecular subtypes with the clinical features and prognosis of HCC

To dissect the potential advantages of the two subtypes identified by consensus clustering applied in actually clinical issues, we compared the correlation between molecular subtypes, various degrees of diverse clinicopathologic characteristics (e.g., such as age, sex), and main prognostic clinical parameters including tumor stage (e.g., T stage, M stage and N stage) and mutation status using Kruskal test with boxplots. Moreover, we assessed the differences in HCC among different subtypes using Kaplan-Meier curves produced by the R packages “survival” and “survminer”.

### Gene set enrichment analysis and gene set variation analysis

As described above, the HCC patients were classified into C1 and C2 subtypes based on CMCRGs. To assess the dominating hallmark genes and signaling pathway relevant with these subtypes, we performed enrichment analysis by uploading the sample groups and gene expression data to Gene Set Enrichment Analysis (GSEA) software (https://www.gsea-msigdb.org/gsea/index.jsp). To verify the different pathway for investigating the divergence in biological function among the two subtypes, we further used the “GSVA” R package to execute the enrichment analysis with the gene sets of “h.all.v7.4.symbols.gmt” downloaded from the MSigDB database. The functional annotation of the CMCRGs were predicted using R package “cluterProfiler”. *P* values < 0.05 were regarded as statistically significant difference in gene ontology.

### Evaluation of tumor microenvironment cells in patients with HCC

Algorithms of immune cell infiltration comprised of CIBERSORT was utilized to compare the immune landscape among different HCC subgroups. Besides, the abundance of immune cell infiltration in different HCC groups was estimated using the single-sample Gene Set Enrichment Analysis (ssGSEA) algorithm in the HCC-LIHC cohort. ESTIMATE software in R package was then used to assess the infiltration extent of immune, and stromal score of each HCC sample (**Table S3**). A series of immune checkpoints and immunomodulator molecules were enrolled for analyzing the immune mechanism (**Tables S4** and **S5**).

### Mutation profile and copy number variation frequency among subgroups

To further validate and characterize the mutation profile, we performed the mutational signature analysis among different subgroups with R software “maftools” package, which is an efficient and comprehensive analysis of somatic variants in cancer and provide visualization process of mutation analysis results. Comprehensive analysis of somatic copy number alteration (SCNA) was detected by the GISTIC 2.0, which has been applied to multiple cancer types.

### Drug-sensitivity analysis and tumor immune dysfunction and exclusion analysis

We predicted the potential sensitivity of chemotherapy or targeted drugs with half-maximal inhibitory concentration (IC_50_) *via* Genomics of Drug Sensitivity in Cancer (GDSC, https://www.cancerrxgene.org) by using the pRRophetic (version 0.5) algorithm in R package. The IC_50_ of each HCC sample was estimated by ridge regression, and the prediction accuracy was evaluated by 10-fold cross-validation according to the GDSC training model. Furthermore, the potential response of immune therapy among different subgroups was estimated with Tumor Immune Dysfunction and Exclusion (TIDE) tool (http://tide.dfci.harvard.edu/), which is a widely used algorithm to predict immune evasion mechanism and immunotherapeutic responsiveness.

### Statistical analyses

All statistical analyses and visualizations were performed with R 3.6.2 and GraphPad Prism v. 8.01 (GraphPad Software, La Jolla, CA, USA). Student’s t test was used to analyze data that satisfied normal distribution. A Mann-Whitney U test was used to evaluate non−parametric data. Kaplan-Meier (K-M) analysis and Cox regression were conducted by the “survival” and the “survminer” packages. The C indices was calculated for each Cox model and pooled using the random-effects mixed model with maximum likelihood. A time-dependent ROC curve was generated by the “timeROC” package to estimate predictive robustness. The “rms” package was employed to fit regression model and depict nomogram. *P* values < 0.05 indicated statistical significance.

## Results

### Identification of CMCRGs subtypes in hepatic carcinoma

**Figure 2:**
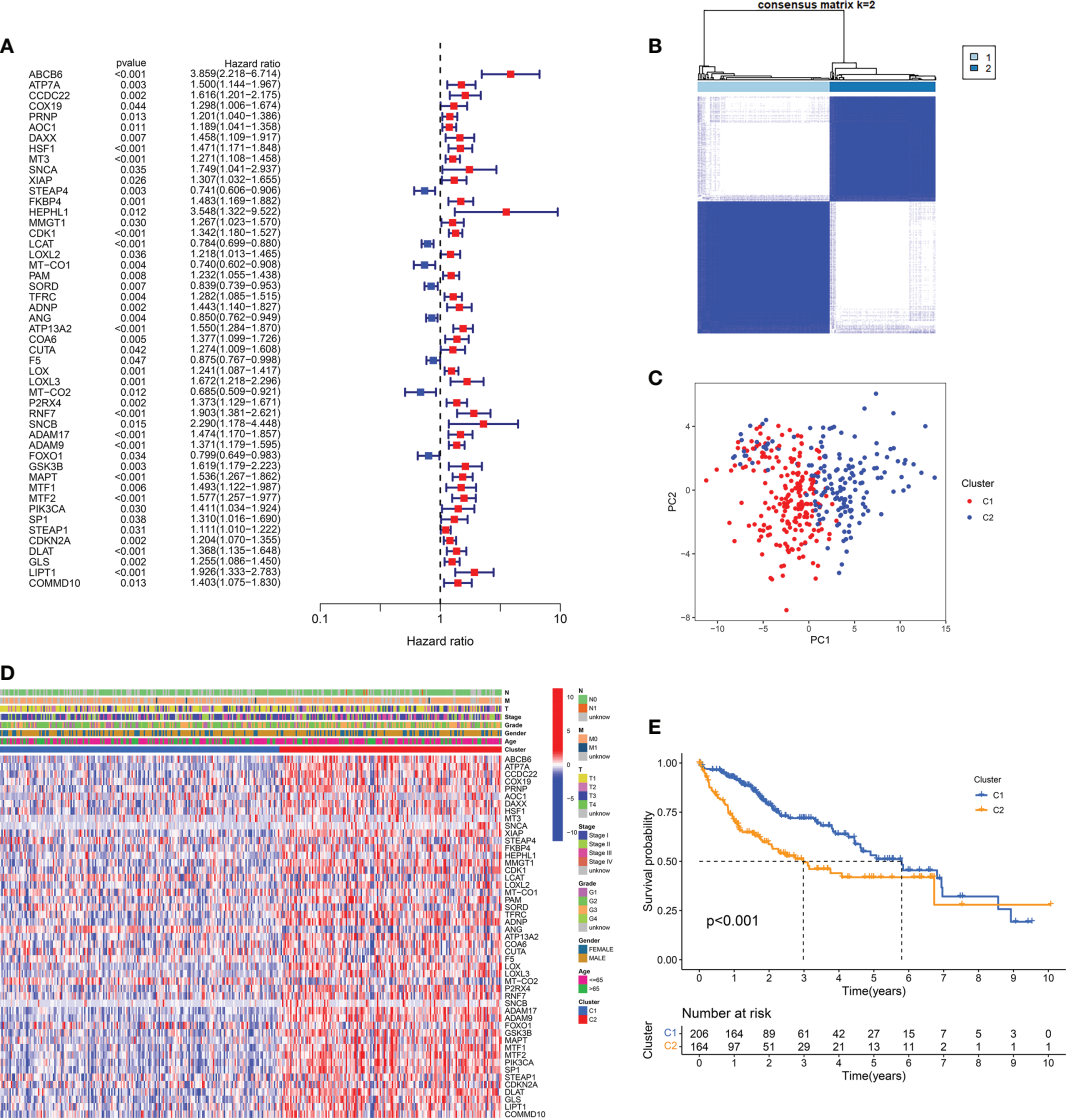
CMCRG subtypes and clinicopathological of two distinct subtypes of samples divided by consistent clustering. **(A)** Forest plot of copper metabolism and cuproptosis-related genes. **(B, C)** Cluster analysis and principal component analysis of two subtypes. **(D)** The expression profile of copper metabolism and cuproptosis-related genes in the two subtypes. **(E)** Survival curve. T: size of the tumor; N: regional lymph node involvement; M: metastasis; Stage: tumor progression stage; Grade: grade of tumor pathological heterogeneity.

The analytical flow of this experiment was shown in **Figure 1**. Through the intersection of the differential expression profiles and the CMCRGs, combined with the survival model simultaneously, a total of 49 genes were screened, which might affect the occurrence and development of hepatic carcinoma (**Figure 2A**). We applied the screened CMCRGs to a cluster analysis of 374 hepatic carcinoma samples, which were divided into two types—C1 and C2 (**Figures 2B, C**). As shown in the heatmap, CMCRGs were mainly highly expressed on C2 subtype (**Figure 2D**). Based on the degree of expression and survival analysis, the C2 subtype was defined as a high correlation group for copper metabolism and had a lower survival rate (**Figure 2E**). We utilized the ICGC database and a self-constructed database for validation. Similar to TCGA, two subtypes based on clustering were significantly discriminated in CMCRGs, which could represent copper metabolism related tumor characteristics for further exploration (**Figure S1A**).

### Clinical characteristics in patients with hepatic carcinoma based on cluster analysis

**Figure 3:**
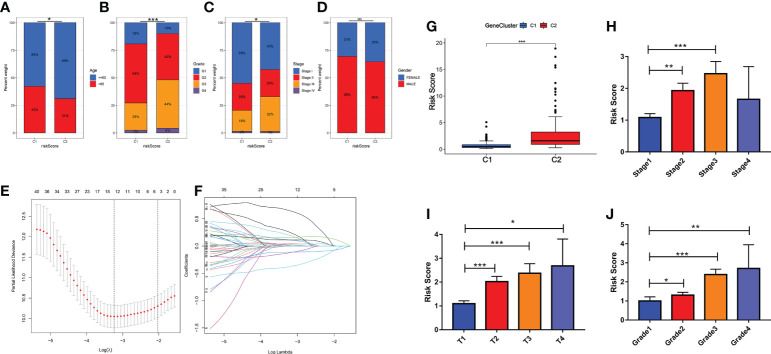
Clinical characteristics in patients with hepatic carcinoma based on cluster analysis. **(A–D)** Analysis of the constituent ratio of clinical characteristics in the two subtypes. **(E, F)** Lasso regression dimensionality reduction on subtype classification results. **(H–J)** Risk scores and pathological characteristics of subtype classification. *P<0.05, **P<0.01, ***P<0.001. NS: Not Significant. G: Grade; T: size of the tumor; Stage: tumor progression stage; Grade: grade of tumor pathological heterogeneity.

The distinction of clinical features in the two subtypes was further analyzed. The significant difference between the two subtypes was mainly focused on age, stage and grade (**Figure 3A-C**). There was no significant difference in gender between the two subtypes (**Figure 3D**). Compared with the C1 subtype, the C2 subtype has higher-grade pathological features. Further, using lasso model dimensionality reduction combined with Cox models, we constructed risk scores based on CMCRGs to characterize both subtypes (**Figures 3E, F**). C2 had a significantly higher risk score than the C1 subtype (**Figure 3G**). The risk scores also had better performance in evaluating different clinical stages. The higher the risk score, the worse the patient’s disease outcome (**Figures 3H-J**). These results suggested that CMCRGs might be associated to the outcome of hepatic carcinoma and therefore have implications for further exploration.

### Gene differences and pathway enrichment between C1 subtypes and C2 subtypes

**Figure 4:**
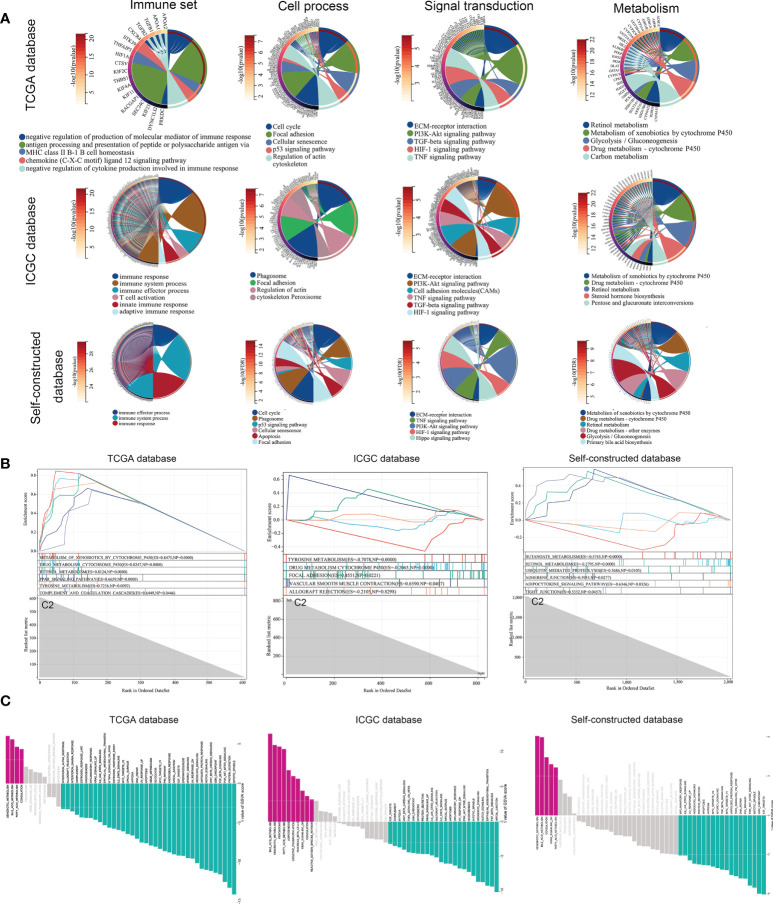
Gene differences and pathway enrichment between C1 subtypes and C2 subtypes. **(A)** Enrichment analysis of immune, metabolic, signaling pathways and cellular processes based on TCGA, ICGC and Self-constructed database. **(B, C)** Enrichment analysis of GSEA and GSVA based on TCGA, ICGC and Self-constructed database.

At the molecular level, we analyzed the difference between the two subtypes and obtained the differential expression profile. Next, an enrichment analysis was performed on these differential genes using four gene sets (metabolism, signal transduction, cell process as well as immunity, etc.). The main differences between subtypes were immune response processes (immune response and immune effector process, etc.), metabolic states (metabolism of xenobiotics by cytochrome P450, etc.), as well as cancer-related signaling pathways (P53 and PI3K-Akt signaling pathway, etc.) (**Figure 4A**). To avoid bias caused by the enrichment analysis ignoring the biological properties of some genes and by differential threshold delineation, GSEA and GSVA analysis were also performed. GSEA analysis showed that there were different immune response and metabolic states between C2 and C1 (‘complement and coagulation cascades’ and ‘tyrosine metabolism’, etc.) (**Figure 4B**). At the pathway level, the results of GSVA analysis also confirmed this conclusion (Xenobiotic metabolism, PI3K-Akt-mTOR signaling and Interferon gamma response, etc.) (**Figure 4C**).

Similarly, we used the ICGC database and a self-constructed database for validation. In both databases, significant differences in immune response (immune response and immune system process, etc.), metabolic status (Metabolism of xenobiotics by cytochrome P450, etc.), and tumor related pathways (PI3K-Akt signaling pathway, etc.) were shown between the two subtypes (**Figures 4A-C**). These results suggested that at the molecular level, copper metabolism and cuproptosis-related genes might be closely related to the immune response and metabolic process in HCC.

### Immune microenvironment analysis of two subtypes

**Figure 5:**
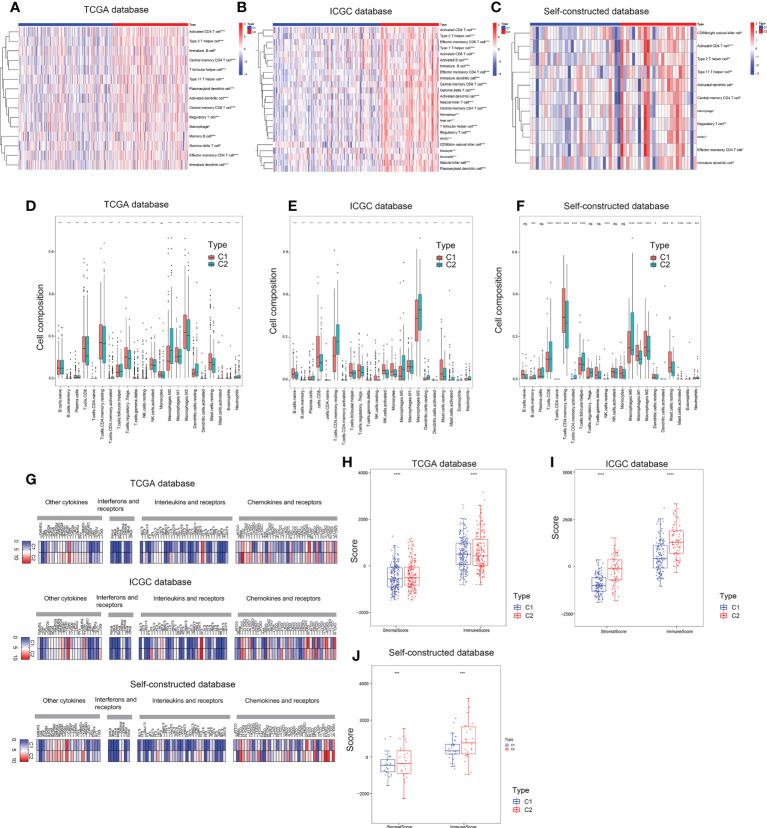
Immune microenvironment assessment of two subtypes. **(A–C)** ssGSEA enrichment analysis based on TCGA, ICGC and self-constructed database. **(D–F)** CIBERSORT analysis based on TCGA, ICGC and self-constructed database. **(G)** The expression profile of key molecules involved in intercellular communication pathways, including cytokines, chemokines and receptors, interferons and receptors, interleukins and receptors based on TCGA, ICGC and Self-constructed database. **(H–J)** ESTIMATE analysis based on TCGA, ICGC and self-constructed database. *P<0.05, **P<0.01, ***P<0.001, ****P<0.0001.

Three different algorithms were used to explore the immune microenvironment between the two subtypes. The TCGA database served as the test set, while the ICGC database and the self-constructed database served as the validation set. CIBERSORT and ssGSEA analyses confirmed that the two subtypes have distinct immune cell composition proportions at the overall level (**Figures 5A-F**). Furthermore, the overall immune cell composition was scored by the Estimate algorithm and showed that the C2 subtype had a higher immune composition score (**Figures 5H-J**).

Furthermore, we analyzed the expression profile of key molecules involved in intercellular communication pathways, including cytokines, chemokines and receptors, interferons and receptors, interleukins and receptors. Compared with the C1 subtypes, cell communication molecules were more highly expressed in C2 subtype (**Figure 5G**). Correlation analysis of communication molecules and immune cells also confirmed that high expression of communication molecules in C2 subtype was closely related to immune cells (**Figure S2A**). These results suggested that copper metabolism status in hepatic carcinoma might have an impact on immunotherapy by affecting the composition of immune cells.

### Analysis of immune checkpoints and tumor immune dysfunction & exclusion in both subtypes

**Figure 6:**
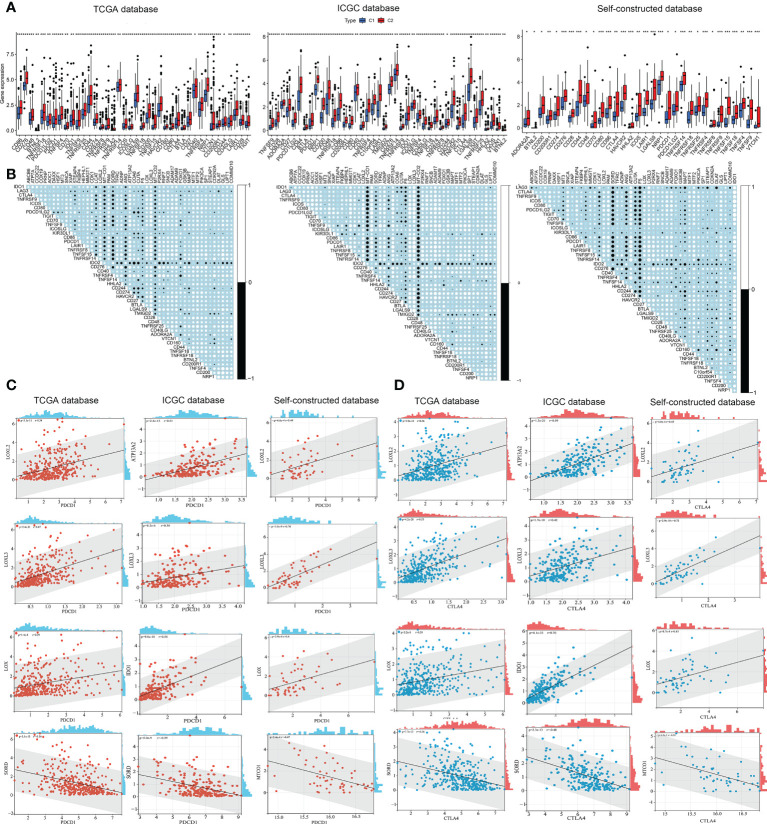
Immune checkpoints analysis in both subtypes. **(A)** Checkpoint analysis among subtypes based on TCGA, ICGC and self-constructed database. **(B)** Correlation analysis between checkpoints and copper metabolism and cuproptosis-related genes based on TCGA, ICGC and self-constructed database. **(C)** Significant correlation genes between PDCD1 and copper metabolism and cuproptosis-related genes based on TCGA, ICGC and self-constructed database. **(D)** Significant correlation genes between CTLA4 and copper metabolism and cuproptosis-related genes based on TCGA, ICGC and self-constructed database. *P<0.05, **P<0.01, ***P<0.001.

Hepatic carcinoma was a typical inflammation-associated cancer, and immunotherapy was an alternative treatment strategy. The landscape of immune checkpoint expression between the two subtypes required further exploration. Immune checkpoint gene expression was significantly higher in C2 subtype compared to C1 subtype (**Figure 6A**). Next, the co-expression correlation matrix of CMCRGs and immune checkpoint genes was used to further clarify the role of CMCRGs on immune checkpoints (**Figure 6B**). Combined with genes previously involved in constructing a risk score, 4 genes (LOXL3, LOXL2, SORD, as well as LOX, respectively) were filtered out, of which LOXL3 had the highest correlation (**Figures 6C, D**). In the analysis of validation set (ICGC and Self), MT-CO1, ATP13A2 and IDO1 were also significantly related to immune checkpoints. LOXL3 had the best concordance among the three datasets (**Figures 6C, D**).

**Figure 7:**
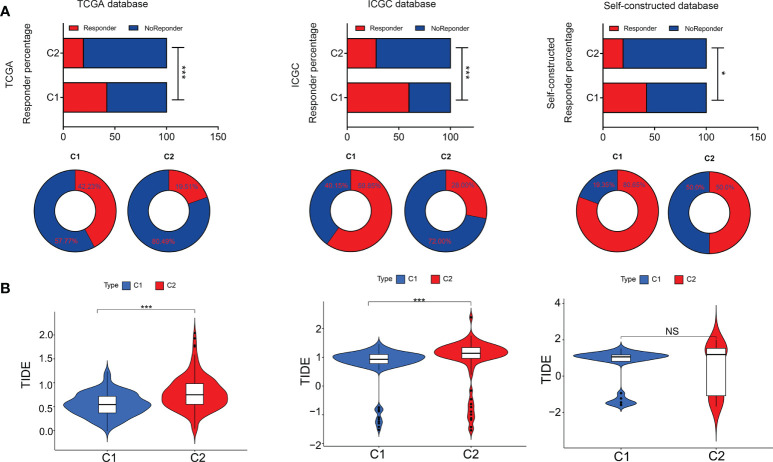
Tumor immune dysfunction & exclusion in both subtypes. **(A)** Responder percentage in Tumor immune dysfunction and exclusion analysis based on TCGA, ICGC and self-constructed database. **(B)** Tumor immune dysfunction and exclusion value between C1 and C2. *P<0.05, ***P<0.001. NS: Not Significant.

In terms of immunotherapy, TIDE analysis (tumor immune dysfunction and exclusion) was used to evaluate the responsiveness of different subtypes to immunotherapy (**Figure 7A**). The results showed that the TIDE score of C2 subtype was significantly higher than that of C1 subtype, and the patients with C2 subtype had lower immune response to immunotherapy (**Figures 7A, B**). These results suggested that C2 subtype was more likely to be closely associated with tumor immune dysfunction and exclusion, thus affecting the efficacy of immunotherapy.

### Identification of subtype mutation characteristics

**Figure 8:**
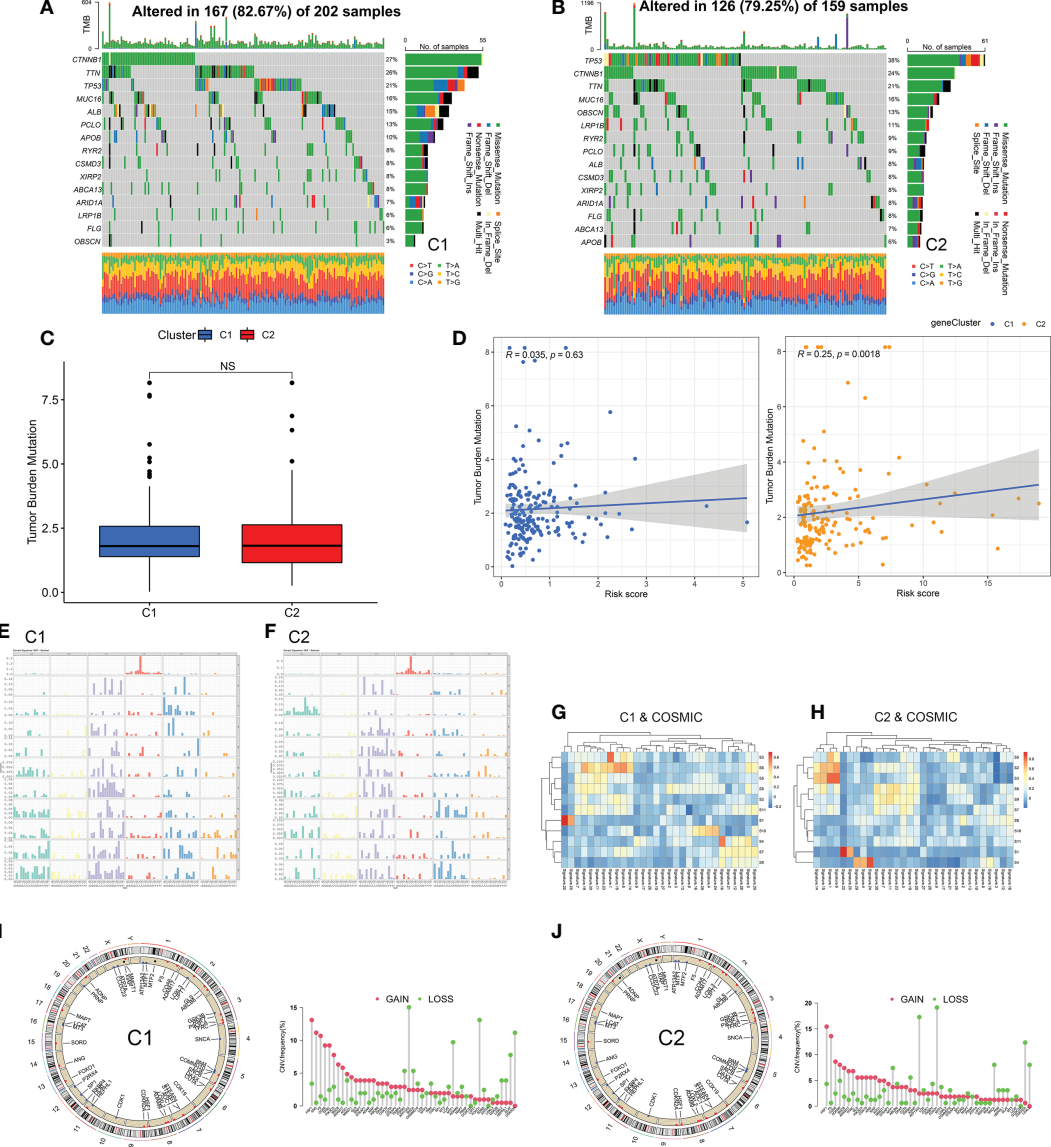
Identification of subtype mutation characteristics. **(A, B)** Immune mutation landscape of driving genes. **(C, D)** Correlation analysis of mutation load and risk score of copper metabolism and cuproptosis-related genes among subtypes. **(E, F)** Frequency spectrum of somatic mutation between subtypes. **(G, H)** The spectrum of somatic mutations between subtypes was analyzed jointly with COSOMIC database. **(I, J)** CNV analysis of copper metabolism and cuproptosis-related genes among subtypes. NS: Not Significant.

In addition to immunotherapy, targeted therapy was another therapeutic strategy. First, we analyzed the driver gene mutation map and found that the mutation frequency of *TP53* and *OBSCN* in C2 subtype was significantly higher than that in C1 subtype (**Figures 8A, B**, and **Figure S3**). In terms of CMCRGs, the results were not statistically significant because single nucleotide mutations occurred less frequently (**Figure S1B**). Meanwhile, there was no significant difference in TMB between the two subtypes (**Figure 8C**). Correlation analysis between the risk score and TMB revealed that the risk score of the C2 subtype, but not the C1 subtype, was significantly associated with TMB (**Figure 8D**).

Next, the mutation characteristics of different subtypes were further analyzed. The direction of analysis was mainly divided into two aspects single nucleotide polymorphisms as well as copy number variations. Based on single nucleotide polymorphism, 96 mutation profiles were constructed and divided into 11 signatures. The somatic signatures of the two subtypes were significantly different (**Figures 8E, F**). Then, the somatic signatures of the two subtypes were annotated using the COSMIC database, and the unique features of the two subtypes were screened out according to the correlation greater than 0.7 (**Figures 8G, H** and **Table 1**). The C1 subtype exhibited an extremely strong transcriptional strand bias for T>C mutations at ApTpN context, with T>C mutations occurring almost exclusively on the transcribed strand. The C2 subtype exhibited a very strong transcriptional strand bias for C>A mutations indicating guanine damage that is being repaired by transcription-coupled nucleotide excision repair.

**Table 1:** Correlation between denovo somatic signatures and cosmic database.

DeconstructSig (C1/C2)	C1 & COSMIC	C2 & COSMIC	C1 & C2 Cor
S1	Signature.22 0.9	Signature.22 0.9	0.949925
S2	Signature.15 0.3	Signature.6 0.7	0.408974
S3	Signature.1 0.7	Signature.1/6 0.8	0.630723
S4	Signature.16 0.3	Signature.24 0.8	0.023968
S5	Signature.11 0.4	Signature.1 0.6	0.086543
S6	Signature.6 0.7	Signature.23 0.5	0.480364
S7	Signature.20 0.4	Signature.5 0.4	0.047464
S8	Signature.19 0.4	Signature.2 0.4	0.321194
S9	Signature.16 0.4	Signature.11 0.5	0.238639
S10	Signature.24 0.5	Signature.16 0.25	0.248606
S11	Signature.20 0.3	Signature.19 0.27	0.251936

In terms of copy number variations, LOXL3 showed a different pattern of mutations in the two subtypes. LOXL3 showed an acquired CNV mutation in the C2 subtype and a deletion CNV mutation in the C1 subtype (**Figures 8I, J**). These results suggest that CMCRGs subtypes might affect the mutation of some specific genes, but would not affect the overall mutation load of tumors.

### Drug sensitivity tests in multiple databases

**Figure 9:**
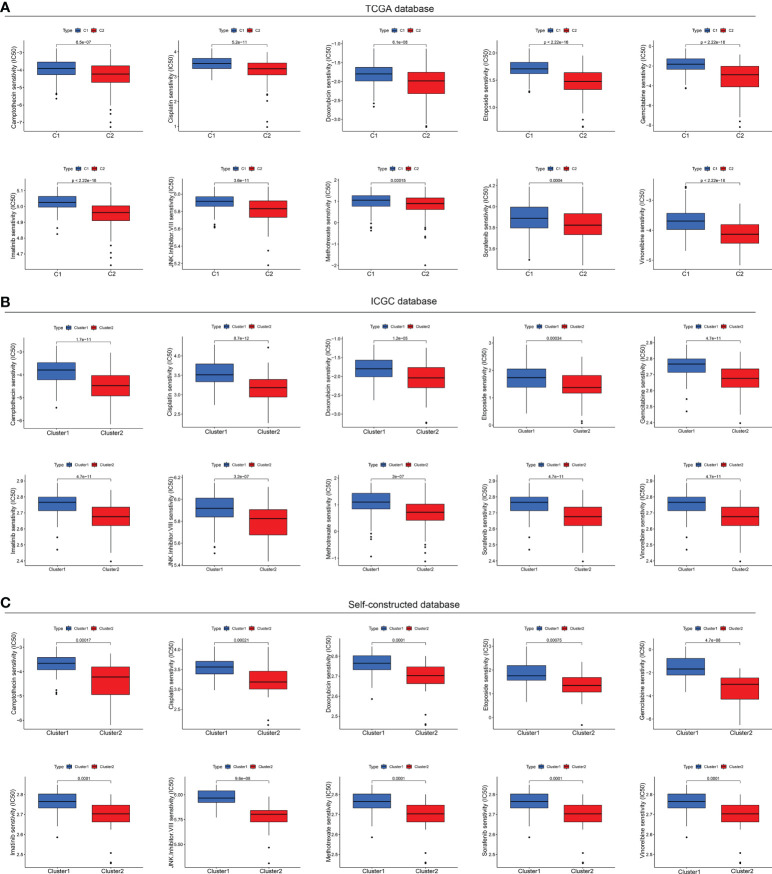
Drug sensitivity tests in multiple databases. **(A)** Drug sensitivity analysis based on TCGA database. **(B)** Drug sensitivity analysis based on ICGC database. **(C)** Drug sensitivity analysis based on Self-constructed database.

Now that the status of CMCRGs might affect the efficacy of immunotherapy, there were also significant differences in the composition of mutations in different subtypes. Furthermore, we evaluated the drug sensitivity responsiveness of drugs to different subtypes of HCC from a tumor drug sensitivity multi omics database. The results indicated that although C2 subtype had lower responsiveness to immunotherapy, it performed better in terms of targeted therapy (**Figures 9A-C**). Specifically, the C2 subtype showed lower IC50 values in camptothecin, cisplatin, doxorubicin, etoposide, gemcitabine, imatinib, JNK inhibitor VIII, vinorelbine, sorafenib and methotrexate susceptibility tests (**Figures 9A-C**). These results would be instructive for subsequent precision treatment.

## Discussion

Due to the strong heterogeneity of HCC, the treatment options for HCC, such as immunotherapy, chemotherapy or surgery are not effective long enough, so that the overall survival rate of patients with HCC is relatively low. Although several studies have shown that molecular subtypes based on immune-related, hypoxia related or ferroptosis-related genes were constructed to guide personalized therapy of HCC, there is still significantly heterogeneity. Therefore, the accurate classification for HCC to improve survival is urgent. Nowadays, several researches have shown that the levels of copper in cancers was significantly increased. In addition, copper also plays a vital role in malignancy biological process of cancers including cell specific death, treatment sensitivity, proliferation, and invasiveness. Moreover, Yang et al. found that commd10, a copper metabolism gene, mediated the radiosensitivity in HCC (21), suggesting that copper also plays an important role in HCC. Thus, genes related copper metabolism and cuproptosis should be concerned and we speculate that the molecular subtypes based on CMCRGs may guide diagnosis and improve survival of HCC.

In the study, we collected 137 CMCRGs from MSigDB and previous studies. Subsequently, we calculated the RNA expression level of the 137 CMCRGs between tumor and adjacent normal tissues in TCGA cohort, and we found that 115 CMCR DEGs, among which 49 genes were associated with prognosis in HCC. Then, we divided HCC from TCGA into two subtypes using 49 prognostic genes through consensus clustering analysis. Notably, compared to C1 subtype, C2 subtype had a poor prognosis, indicating that CMCRGs in HCC are heterogeneous and that the survival outcome was significantly different between different CMCRGs molecular subtypes.

Additionally, the survival outcome was significantly different between the two molecular subtypes, which clarified the role of CMCRGs in HCC prognosis. Then, ORA (Over-Representation Analysis), GSEA and GSVA were conducted to find the pathways between C1 and C2. The genes that have been differentially modulated are associated with pathways, including p53 signaling pathway, PI3K-Akt signaling pathway, HIF-1 signaling pathway, Hippo signaling pathway, TNF signaling pathway, chemokine ligand 12 signaling pathway, and cytokine production; moreover, retinol metabolism, carbon metabolism, glycolysis, gluconeogenesis, and primary bile acid biosynthesis related with cancer also changed in the two clusters. Previous studies had showed that p53 signaling pathway, PI3K-Akt signaling pathway, HIF-1 signaling pathway are important pathways involved in tumor development and metastasis (22, 23). Additionally, glycolysis, gluconeogenesis, and bile acid biosynthesis also play an important role in the tumor progression (24–26). Moreover, immune signaling pathways, including TNF signaling pathway, chemokine ligand 12 signaling pathway, and cytokine production, act a critical role in tumor (27, 28). These results were validated in the dependent own data and ICGC-JP. Therefore, the pathways gave a hint of the importance of immune between the two molecular subtypes.

However, the relationship is greatly complex between tumors and immune cells. Previous studies have reported that M2 macrophage promote tumor invasion, progression and immune escape through increasing the expression of MHC class I molecules and secreting anti-inflammatory cytokines (29–31). Moreover, Tregs also inhibit the immune response, which led to immunosuppressive functions and accelerated the malignant tumors progression (32, 33). Neutrophils secrete some factors, such anti-inflammation cytokines, to affect TME, promoting tumor occurrence and metastasis (34, 35). Additionally, DCs, natural killer (NK), natural killer T (NKT) cells and CD8^+^ T cells act as strong cytotoxic functions through secreting pro-inflammatory cytokines, which promote tumor immunosuppression (36–39). Thus, ssGSEA, CIBERSORT and ESTIMATE analysis were used to analyze immune infiltration to estimate the activities of TME cells in HCC. The results showed that the abundance of activated B cell, M1 macrophage cell, and activated CD8^+^ T cell, which were the main pro-inflammatory cells were high in C1. And C2 contain the immunosuppressive cell, including M2 macrophages, Tregs, neutrophils and so on. These results suggest that different clusters had critical differences in the cellular component. Moreover, C1 with effective antitumor immune cells had a better survival than that in C2, which was in consistent with previous study (40). Though the infiltration of immune cells regulates immune activity and improve the prognosis, the therapeutic effect in various cancers was not as expected. Therefore, it is important to focus on the immune checkpoints (ICB), which also affect the prognosis. Then, the differences of the expression in ICBs between C1 and C2 were explored. The results showed that the expressions of inhibitory immune checkpoint, such as PD-1, CTLA-4, LAG3, TIM3 and TIGIT, in C2 were increased compared to those in C1, which hinted that HCC patients in C1 might be better responsive to immunotherapy. Various researches came to the same conclusion. For example, Chuah et al. reported that anti-PD-1 drug significantly improves the response for HCC and offers mechanistic insights into the immune trajectories in different immune subsets, indicating that immunotherapy targeting inhibitory immune checkpoints is promising in HCC (41). Wan et al. showed that MTDH could improve the anti-PD-1 response and increase cytotoxic T-cell infiltration, indicating that the effectiveness of MTDH for predicting immune checkpoint inhibitor treatment in HCC (42). Meanwhile, TIDE analysis showed a higher proportion of patients with immune tolerance in the C2 subtype. Based on the results of these analyses, we believed that patients in the C2 subtype might not have optimal outcomes when receiving immunotherapy regimens.

On the other hand, some studies also reported that the responsiveness or tolerance to immunotherapy was significantly related with tumor mutations (43, 44). Thus, we analyzed the mutation profiles and found that the TMB between C1 and C2 was similar. However, the oncogenic driver genes, including *TP53* and *OBSCN*, were frequently changed. In this study, we observed that the *TP53* mutation in C2 was higher than that in C1. It is well known that *TP53* regulated tumor progression and metastasis through several pathways including cell apoptosis, proliferation and cancer stem cells. Moreover, the mutations of *TP53* indicate the poor prognosis in various cancers, including HCC. Interestingly, *OBSCN*, a new oncogene, significantly mutated in C2. Various studies had shown that the mutation of *OBSCN* is strongly associated with cancers (45–47). This is consistent with the result that C2 had a poor prognosis.

It is well known that HCC is mainly treated by surgery, immunotherapy and chemotherapy, and is usually resistant to immunotherapy. However, chemotherapy drugs can turn the cold tumor environment of patients to hot ones so that to improve the effect of immunotherapy. Thus, it is of great importance to understand the chemosensitivity of HCC CMCRGs clusters. In our research, C2 were more sensitive to camptothecin, cisplatin, doxorubicin, etoposide, gemcitabine, imatinib, methotrexate, and sorafenib. Thus, the identification of CMCRGs subtypes are conducive for chemotherapy optimization and improve the curative effect in HCC.

However, there are several limitations in our study. Firstly, data from public databases and our data, was small and the clinical follow-up information was lack, which may cause a selection bias, affecting the results. Therefore, a large HCC samples need to be enrolled. Secondly, the results were obtained by bioinformatics analyses. Thus, we should conduct experimental studies to validate these results. Thirdly, some clinical variables, such as chemoradiotherapy, neoadjuvant chemotherapy, were unavailable, which influence the robustness of immunotherapy efficacy. Moreover, large immunotherapy cohorts are needed for predicting prognosis and immune response.

## Conclusions

In conclusion, we characterized the two subtypes of HCC based on copper metabolism and cuproptosis-related genes, and found that different subtypes had distinct prognosis. Moreover, the differences in signaling pathways and immune networks between the two subtypes in HCC were explored, which provided more insights between copper metabolism and cuproptosis and immunity. Thus, our study suggested that the subtypes based on copper metabolism and cuproptosis-related genes might be helpful to proposed novel insights and guided clinical treatment strategies for HCC.

## Data availability statement

The datasets presented in this study can be found in online repositories. The names of the repository/repositories and accession number(s) can be found in the article/**Supplementary Material**.

## Ethics statement

The studies involving human participants were reviewed and approved by Zhengzhou Central Hospital Affiliated to Zhengzhou University. The patients/participants provided their written informed consent to participate in this study.

## Author contributions

XL, XZ, and JJ designed the study. XL, BS, and JJ collected study data, XL, BS, and JX performed computational analysis. XL, XZ, and JJ wrote the manuscript draft. YY, LL edited the manuscript draft. YY, XW revised the draft. All authors have read and approved the manuscript.

## Funding

This study was supported by funds from the National Natural Science Foundation of China (No.81870983) and Scientific and Technological Innovation Action Plan of Shanghai (No.22140902200).

## Conflict of interest

The handling editor XH declared a shared parent affiliation with the authors XL, XW, XZ at the time of review.

The remaining authors declare that the research was conducted in the absence of any commercial or financial relationships that could be constructed as a potential conflict of interest.

## Publisher’s note

All claims expressed in this article are solely those of the authors and do not necessarily represent those of their affiliated organizations, or those of the publisher, the editors and the reviewers. Any product that may be evaluated in this article, or claim that may be made by its manufacturer, is not guaranteed or endorsed by the publisher.
