# Supplementary material for: Identification of copper metabolism and cuproptosis-related subtypes for predicting prognosis tumor microenvironment and drug candidates in hepatocellular carcinoma
Source: Front Immunol. 2022 Oct 6;13:996308. doi: 10.3389/fimmu.2022.996308 (PMC9582144; doi:10.3389/fimmu.2022.996308)
Supplement: Supplementary file 1 [file DataSheet_1.docx]

***Supplementary Material***


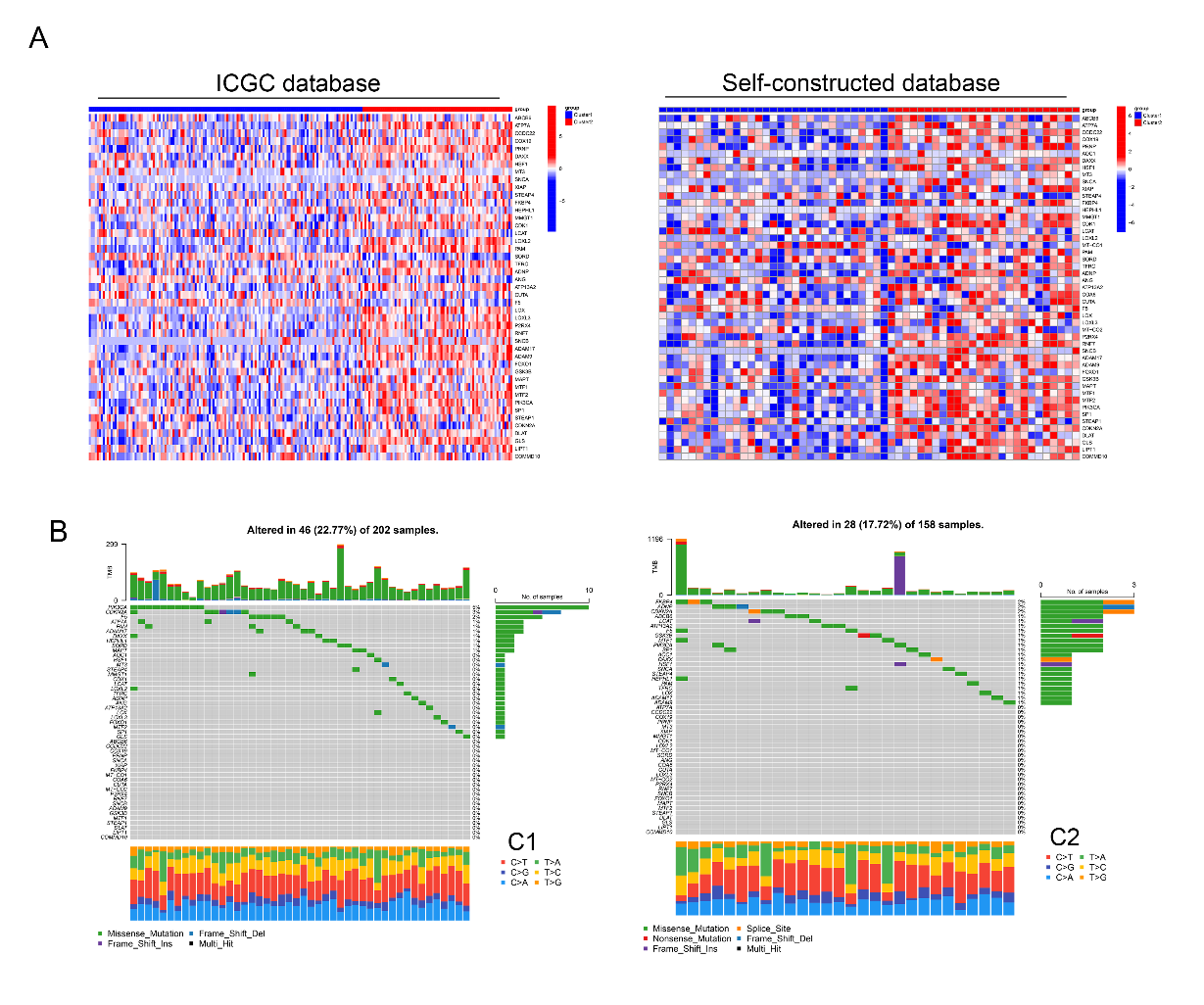


**Supplementary Figure 1. Clustering and mutation analysis.** **(A)** The expression profile of copper metabolism and cuproptosis-related genes based on ICGC and self-constructed database. **(B)** The mutation landscape of copper metabolism and cuproptosis-related genes in the two subtypes.


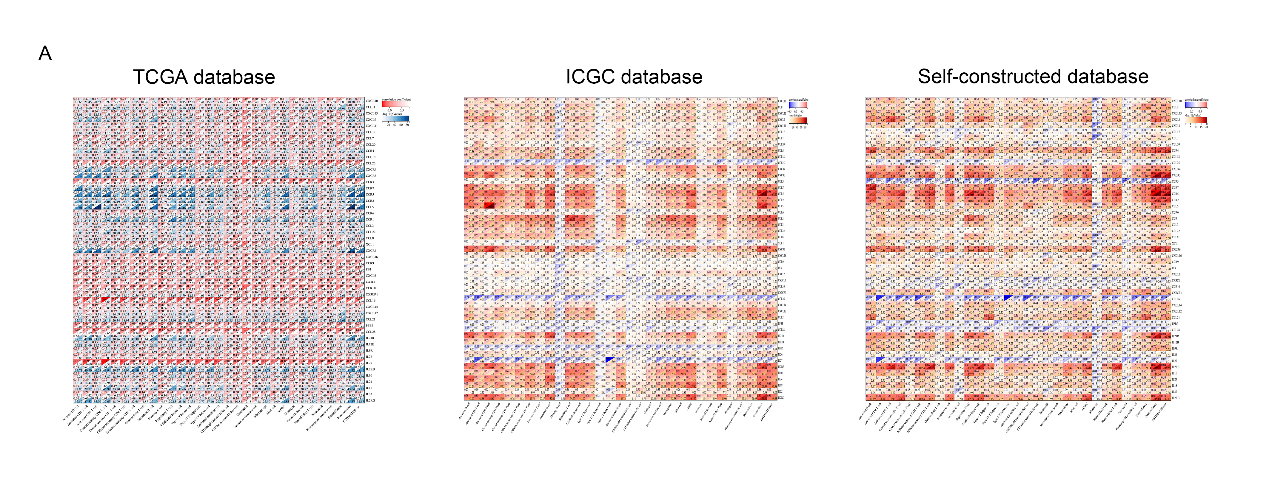


**Supplementary Figure 2. Correlation analysis. (A)** Correlation analysis of communication molecules and immune cells based on TCGA and self-constructed database.


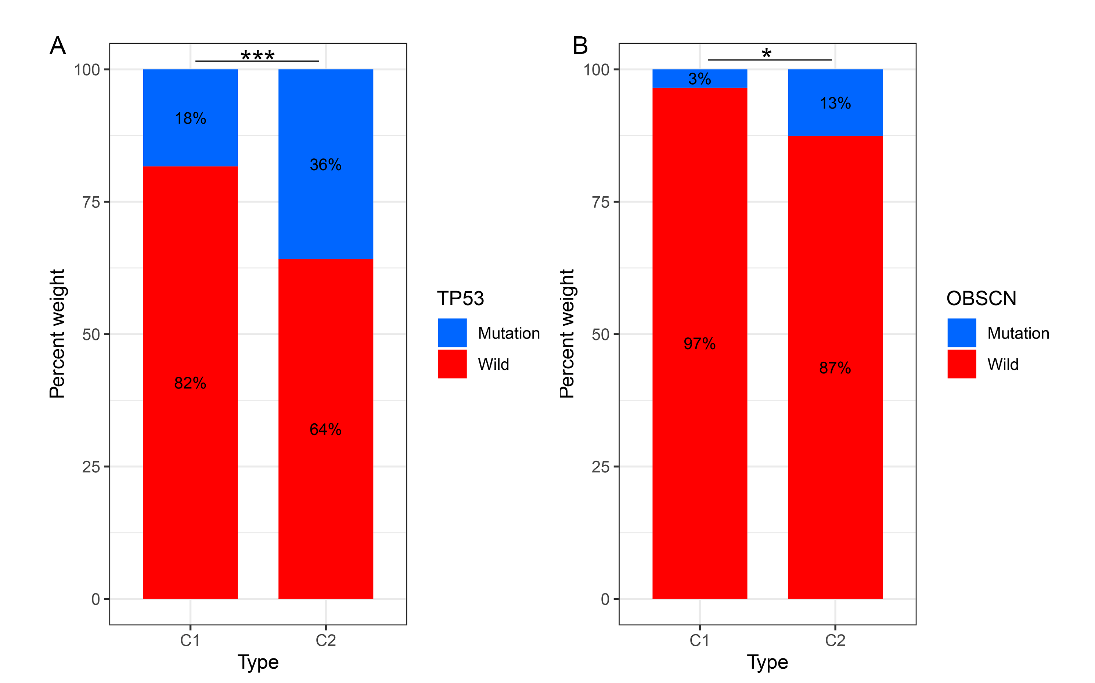


**Supplementary Figure 3. Analysis of mutation composition ratio of two subtypes.** (**A**) Mutation of TP53 between the two subtypes. (**B**) Mutation of OBSCN between the two subtypes.
